# Mechanistic and Machine Learning Modeling of Microwave Heating Process in Domestic Ovens: A Review

**DOI:** 10.3390/foods10092029

**Published:** 2021-08-29

**Authors:** Ran Yang, Jiajia Chen

**Affiliations:** Department of Food Science, University of Tennessee, Knoxville, TN 37996, USA; ryang17@vols.utk.edu

**Keywords:** microwave heating, temperature uniformity, mechanistic modeling, machine learning, product design

## Abstract

The domestic microwave oven has been popularly used at home in heating foods for its rapid heating rate and high power efficiency. However, non-uniform heating by microwave is the major drawback that can lead to severe food safety and quality issues. In order to alleviate this problem, modeling of microwave heating process in domestic ovens has been employed to simulate and understand the complicated interactions between microwaves and food products. This paper extensively reviews the mechanistic models with different geometric dimensions and physics/kinetics that simulated the microwave heating process. The model implementation and validation strategies related to the model accuracy and efficiency are also discussed. With the emergence of the machine learning technique, this paper also discusses the recent development of hybrid models that integrate machine learning with mechanistic models in improving microwave heating performance. Besides, pure machine learning models using only experimental data as input are also covered. Further research is needed to improve the model accuracy, efficiency, and ease of use to enable the industrial application of the models in the development of microwave systems and food products.

## 1. Introduction

Microwave ovens have been a popular domestic appliance for their convenience, rapid heating rate, and high power efficiency. Over 95% of households in the U.S. own at least one microwave oven [1] and mainly use it for cooking, defrosting, and re-heating [2]. The fast-heating characteristic of microwave heating can help retain heat-sensitive compounds in foods, and therefore keep the color, texture, flavor, and nutrients [3,4]. The advantages of microwave heating are mainly attributed to its energy delivery through electromagnetic radiation, where the microwaves penetrate into the foods and agitate the polar molecules and charged particles to generate heat volumetrically [5]. However, this advantageous heating method of microwaves has a non-uniform heating issue, which may cause severe food quality and safety problems. Microwave-heated food is often considered low-quality food due to the uneven distribution of hot and cold spots [6,7], which degrades consumers’ experience. The overheating and high moisture loss lead to significant quality loss at the hot spot areas; and conversely, the cold spots cannot be thoroughly heated to ensure food safety [8,9]. Several foodborne outbreaks related to microwaveable food products have been reported in the past years [10].

The non-uniform microwave heating can be attributed to the complicated interactions between microwave and food products, which are influenced by many oven- and food-related factors. Previous studies showed that the oven cavity designs, shape and position of the foods [9], dielectric [11] and thermal properties [12] of the food materials, are all factors that influence the microwave-food interactions. The domestic microwave oven cavity is a multimode cavity, where the electric field is not uniformly distributed within the cavity and food product [13]. The high and low electric field density and microwave power distribution within the cavity are generally fixed during the heating process, known as standing wave patterns [14]. The standing wave pattern leads to the non-uniform heating of food products. In addition, the non-uniform level is exaggerated over the microwave heating process, especially for frozen food products, which is known as the “thermal runaway” effect. The “thermal runaway” phenomenon is caused by the temperature-dependent changes of dielectric and thermal properties of food products. At frozen state (or low temperatures), food products often have a relatively lower dielectric loss factor than those at thawed state (or high temperatures) [15]. Due to the non-uniform distribution of the microwave power, part of the food products will be thawed first (or heated more), which raises the dielectric loss factors and, thus, the microwave energy absorption in those regions. The consequence is that the hotter regions of the food product absorb more and more microwave power. Additionally, the latent heat of melting in frozen food products worsens the “thermal runaway” effect [16]. 

Based on the existing knowledge about microwave heating, various strategies that aim to improve the heating results were proposed and demonstrated, and several of them have already been applied to the domestic oven designs. For example, a turntable is often used in commercial domestic microwave ovens to increase the heating uniformity, where the improvement was reported to be up to 43% [17]. However, the improvement is only achieved in the circumferential direction while not along the diametral direction [18]. Another approach is that different types of the mode stirrers, such as single propeller-shaped mode stirrer [19], double propeller-like stirrers in front of the waveguide ports that are located on the opposite sidewalls [20], double plate-shaped mode stirrers vertically attached to the top wall [21], and rotary disk mode stirrers [22], can be incorporated into the microwave ovens to improve the microwave heating uniformity. Furthermore, assembly of a mode stirrer under the turntable was also proposed and demonstrated to improve temperature uniformity [23,24]. 

Besides the modification on the microwave oven cavity, the properly designed food products and packages can also improve the microwave heating performance. In microwave heating of foods, the penetration depth is one critical parameter that is defined as the depth in foods at which the microwave power drops to 1/e of its initial value at the surface [25]. The penetration depth is usually used as a reference to determine the thickness of a food product for optimal microwave heating performance [26]. In addition, metal shielding [27] and steam venting [28,29,30] were also used in microwavable food packages to improve heating uniformity. 

Traditionally, trial-and-error microwave heating experiments are widely used in designing microwave ovens, food products, and packages. However, due to the complicated interactions among microwaves, food, and packages, it is difficult to observe and understand the complicated interactions experimentally, especially for the novel approaches (e.g., new package designs) that complicate the interactions where various physical, chemical, and biological processes are involved. Hence, it is critical to deeply understand these complicated interactions, which can guide future food product development strategies. In the past decades, a variety of mathematical models for simulating the microwave heating process have been developed as promising tools to understand the complicated interactions but have not been extensively adopted by the industry in the designs of ovens, foods, and packages due to significant limitations of model accuracy, efficiency, and ease of use. Besides, there is an increasing trend to apply or couple machine learning skills to either replace or assist the basic mechanistic models, which has been used in microwave heating process and also raised extensive discussions in biology [31]. The novel strategy shows its advantages in computation power while cannot fully overcome the limitations due to lack of knowledge on the microwave-food interactions during the heating process.

The purposes of this review are to (1) review and discuss various mechanistic models based on different geometric dimensions and physics/kinetics that simulated the heating process in domestic microwave ovens, (2) review the recent development of using machine learning techniques in microwave heating modeling, and (3) summarize current models and discuss the future aspects in improving the model accuracy and efficiency and developing them as easy-to-use food/oven development tools for industrial implementation.

## 2. Mechanistic Models

Microwave heating of food in domestic ovens is a complicated process that involves multiple physics, chemical reactions, and biological dynamics. During the heating process, the electromagnetic wave at around 2.45 GHz is generated in the magnetron and fed into the oven cavity through a waveguide. Part of the microwave energy is absorbed by the food product and converted into thermal energy to heat the food products volumetrically. Due to the non-uniform power distribution, hot and cold spots are generated within the food product, which results in temperature gradient and heat transfer. As the temperature increases, moisture evaporation occurs and generates high pressure at hot spots. The pressure gradients and moisture gradients drive the moisture movement within the food product, which also promotes the heat transfer process. The significant moisture loss and pressure-driven flow may also have a mechanical effect, which causes geometry change of the food product. Along with the temperature and moisture change, chemical reactions of color change and nutrients degradation also occur during the microwave heating process, which affects the quality of the microwaved foods. Meanwhile, the microorganisms are also inactivated dynamically with time, which is related to product safety. Thus, in order to develop microwaveable food products with high quality and safety, it is necessary to understand the complicated physical process, chemical reactions, and microbial inactivation during the microwave heating process.

Many mechanistic models have been developed to study the complicated physical, chemical, and microbial processes with various dimensional and physics/kinetics considerations. The early models start with significantly simplified model geometry (e.g., one-dimensional, 1-D geometry) and mechanistic physics (e.g., only electromagnetics). There are also complex models in low dimension but with comprehensive physics to estimate heat and mass change [32,33]. The most recent models can simulate the microwave heating process using detailed 3-D oven and food geometries, as well as comprehensive physical, chemical, and microbiological dynamics. These models have divergent capabilities of predictions and require different model inputs and computational resources, as summarized in Table 1.

### 2.1. One-Dimensional Models for Simulation of Energy Conversion

Early-stage simulations of the microwave heating process assumed the food product as a 1-D line (e.g., sphere sample). The microwaves were fed from one side of the product (a point) and only propagated in one direction along the line; the electromagnetic power was dissipated and used to heat the food product.

When the dielectric properties are constant, the generated heat from electromagnetic power could be expressed in closed-form based on Lambert’s law. Several analytical models have been developed to predict the temperature distribution and reveal the non-uniformity problem along the propagation direction of the microwave [34,36,37,56]. For example, a 1-D model proposed by Campañone and Zaritzky [34] coupled electromagnetic heating, following Lambert’s law, and conductive heat transfer to simulate the microwave heating of sphere-shaped food. Minced beef was used as a model food, and constant material properties were used in this study. The Lambert’s law applied in this model assumed that microwave absorption within the products exponentially decayed with distance, which was expressed as:

(1) $$q=q_{0}e^{-2\alpha d}$$

where q is the dissipated power, $q_{0}$ is the surface power, *d* is the maximum distance measure from the surface, and *α* is the attenuation factor expressed by Equations (2) and (3):

(2) $$\alpha =\frac{2\pi }{\lambda }\sqrt{\frac{\varepsilon \prime \left({\left(1+tan^{2}\delta \right)}^{\frac{1}{2}}-1\right)}{2}}$$

with

(3) $$\delta =tan^{-1}\left(\frac{\varepsilon \prime \prime }{\varepsilon \prime }\right)$$

where λ is the wavelength of the microwave in free space, ε’ is the dielectric constant, ε” is the dielectric loss factor, and tanδ is the loss tangent. 

The conductive heat transfer was described by Equation (4):

(4) $$\rho C_{p}\frac{\partial T}{\partial t}=\nabla \left(k\nabla T\right)+q$$

where ρ is the density, and $C_{p}$ is the thermal capacity, *k* is the thermal conductivity, *t* is time, and *T* is temperature.

This 1-D model was solved by the finite difference method (FDM) and predicted the heating profile along one line. The model results showed good agreement with experimental point temperatures in the sphere center and on the surface of the beef sample, with a relative error of −2.46%, after 48 s microwave heating under 2.45 GHz.

Another 1-D model developed by Kopyt and Celuch-Marcysiak [35] simulated the microwave heating process. Instead of using Lambert’s law, this model described the electromagnetic power by Maxwell’s equations and solved the model by FDM. Maxwell’s equations in the differential form are as Equations (5)–(8):

(5) $$\nabla \times E=-\frac{\partial B}{\partial t}$$

(6) $$\nabla \times H=J+\frac{\partial D}{\partial t}$$

(7) $$\nabla \cdot D=\rho_{e}$$

(8) ∇·B=0

where ***E*** is the electric field, ***B*** is the magnetic induction, ***H*** is the magnetic field, ***J*** is the current flux, ***D*** is the electric displacement, and $\rho_{e}$ is the charge density. And with the solved electric field, the power source for the heat transfer along the 1-D line direction was calculated following Equation (9):

(9) $$q\left(x,t\right)=\frac{1}{2}\omega \varepsilon_{0}\varepsilon^{\prime \prime }{\left|E\left(x,t\right)\right|}^{2}$$

where $\varepsilon_{0}$ is the permittivity of free space, ω is the angular frequency of the microwave and $E\left(x,t\right)$ follows:

(10) $$E\left(x,t\right)=E_{0}e^{-\alpha_{0}\left(t\right)x}$$

where ***E_0_*** is an arbitrarily assumed initial amplitude of the electric field, $\alpha_{0}\left(t\right)$ is the real part of the time-dependent propagation constant of the microwave. This model was solved based on a steady-state electromagnetic field, and the obtained electric field $E\left(x,t\right)$ was used as the power source to solve the source function $q\left(x,t\right)$ by Equation (9). Through the simulation process, the dielectric properties of the materials were assumed to be constant. The time-dependent process was simulated until the end of the heating time. The results of this model could predict the dynamic microwave heating temperature with various total heating times. 

Zhong et al. [36] improved these 1-D models by comprising temperature-dependent physical properties of density, specific heat capacity, and thermal conductivity in the heat transfer equation (based on Equation (4)), which could predict the temperature profiles that reflect more details. Another 1-D analytical model further incorporated more details by including the electromagnetic wave reflection to simulate the microwave heating inside an oven cavity [37]. This model assumed that the food product was placed on a glass turntable inside an oven cavity and three material layers of food, glass turntable, and air gap between glass and metal oven bottom. The planar waves were assumed to propagate vertically from the top surface of the food to the oven bottom and reflected back to the food product. The superimposed forward and reflected electromagnetic waves were used to calculate the total microwave power absorption and the average heating rate of food products. The established 1-D model was applied to determine the optimal thicknesses of two compartments of a food product for better heating uniformity. The model was validated by heating three model food systems consisting of two compartments (water-butter, pasta-sauce, and sauce-butter systems), and the validation results demonstrated the usefulness of the model.

### 2.2. Two-Dimensional Models for Simulation over Planes

Since in 1-D simulation, the power only propagates along one direction, and the 2-D spatial heating performance cannot be expressed. In order to estimate the non-uniform distribution of temperature in both vertical and horizontal directions, 2-D models were developed to predict the power distribution over selected planes. Expanding on the 1-D models, several studies used 2-D models to understand the interactions between electromagnetic waves and food products and evaluated the spatial temperature distributions. Pandit and Prasad [38] developed 2-D models to predict the transient temperature of both the cylinder- and slab-shaped potato samples by coupling Lambert’s law and conductive heat transfer. For the slab-shaped sample, a plane slice (x-y) was used to represent the product, and the electric field was assumed to attenuate exponentially in the x and y directions within the food product. For the cylinder-shaped product, the radical coordinate of radius-length (r-z) was used to represent the product, and the electric field was assumed to attenuate exponentially in the r and z directions within the food product. The models were validated and used to evaluate the effect of sample size on microwave heating performance. The results identified high microwave power distribution at the corner of the slab-shaped sample, which was due to the net absorption of microwave power from two adjacent surfaces forming the corner, and a high concentration of absorbed power along the central line of the cylinder, which was attributed to the rapid increase of power density near the center.

Similarly, Liang et al. [39] developed a transient 2-D model to evaluate uneven microwave heating. In this study, a slice (x-y) of deionized water was fully filled in a rectangular resonant cavity, and uniform microwave power was perpendicularly applied from one side (y) and propagated along the x-direction. Both the forward and backward traveling waves were incorporated in the model based on Lambert’s law to calculate the microwave power dissipation. Transient conductive heat transfer was used to simulate the temperature distribution. Moreover, the wave reflected from the oven walls and generated standing wave patterns, where the oven geometry was partially considered in the simulation. The transverse electric fields of the resonant cavity were expressed with sinusoidal:

(11) $$E_{t}=A^{+}\sin\left(\frac{\pi }{b}x-\omega t\right)+A^{-}\sin\left(\frac{\pi }{b}x+\omega t\right)$$

where $A^{+}$ and $A^{-}$ are arbitrary amplitudes of the forward and backward traveling waves, *b* is the width of the oven cavity and *x* is the variable for longitudinal position along the wave propagation direction. 

The spatial heat distribution function was developed based on Equation (4), and was defined as:

(12) $$\begin{array}{c} \rho \left(T\right)C_{p}\left(T\right)\frac{\partial T}{\partial t}-k\left(T\right)\left(\frac{\partial^{2}T}{\partial x^{2}}+\frac{\partial^{2}T}{\partial y^{2}}\right)=q\left(x,y,t\right)\\ \;\;\;\;\;\;\;\;\;\;\;\;\;\;\;\;\;\;=q_{max}{\left[e^{-\frac{x}{D_{p}}}\sin\left(\frac{\pi }{a}x-\omega t\right)+e^{-\frac{2l-x}{D_{p}}}\sin\left(\frac{\pi }{a}x+\omega t\right)\sin\left(\frac{\pi }{a}y\right)\right]}^{2} \end{array}$$

where $D_{p}$ is penetration depth, l is longitudinal distance.

The dynamic power distribution within the sample plane could be simulated by this 2-D model. The model results showed that uniform distribution of temperature in the early stages of the heating, but the temperature difference increased with time, and hot spots appeared inside the sample. With the capability of predicting time-dependent regional temperature, especially the hot and cold areas, this model was also proposed to serve as part of the optimization algorithm to improve the microwave heating performance.

Microwave oven with mode stirrers, usually in the form of mobile metabolic sheets, was simulated with a 2-D model by Plaza-González et al. [7]. The simulated plane was vertical to the bottom wall and included the power input through a top waveguide, two laminated mode stirrers on the two sides of the port on top, and a layered sample placed in the center of the bottom. The electric field was calculated as:

(13) $$E_{mean}\left(x,y\right)=\sqrt{\frac{\sum_{i}^{N}E_{i}^{2}\left(x,y\right)}{N}}$$

where $E_{mean}$ is the average electric field, $E_{i}\left(x,y\right)$ is the instantaneous spatial field distribution within the sample for position *i* of the mode stirrers, and *N* is the total number of different simulated stirrer positions. With the oven cavity considered in this 2-D model, the interactions among waves, oven geometry, and the mode stirrers could be analyzed. The dynamic distribution of the electric field due to the rotation of stirrers simulated by this model showed good agreement with experimental results but was different from the models that only used Lambert’s law. While when the oven was equipped with mobile parts that lead to nonstationary waves, the model based on average electric fields has better prediction performance.

### 2.3. Three-Dimensional Models for Comprehensive Simulation and Analysis 

With the advancement of computational powers (e.g., high power computational workstations and commercial simulation packages, etc.), more comprehensive 3-D models are now widely used and endowed with the power to incorporate more physics/kinetics, which allows more accurate simulation and prediction for scientific research. Given the enhanced computational power, the detailed geometric information of the food materials, packages, oven cavity, and the relative locations of these elements can be incorporated in the 3-D models. The simulation of dynamic changes of power and temperature in 3-D space by these models could provide more visual clues for the interactions during microwave heating, making the modeling tools easily accessible to both scientific researchers and food manufacturers. To study the interactions among all potentially contributing physics/kinetics, the precise selection of the physics/kinetics and proper simplification are equally critical for the model establishment.

#### 2.3.1. Electromagnetic Heating 

Similar to the lower-dimensional models, the basic physics that are needed in the 3-D multiphysics models are electromagnetics and heat transfer. A fundamental electromagnetic heating model implemented by Geedipalli et al. [17] coupled electromagnetic and heat transfer physics to simulate a 35-second heating process in a domestic microwave oven to evaluate the effect of rotating turntable on the heating uniformity. The microwave propagated from a four-prism-shaped waveguide which is assembled to the sidewall of the oven cavity. The input power was determined experimentally by measuring the power absorption in heating a container of water. The measured power was directly applied to the assumed rectangular port, which was the side face of the waveguide. This model used only constant material properties due to slight temperature change within the short heating process. In order to mimic the rotation of the turntable, the rotational circle was taken as 24 discrete locations, and each one was solved individually. The spatial temperature profiles were updated after each discrete step. Based on the model results, a better uniformity was achieved by the use of the turntable, where the overall improvement was around 40%.

The two-physics model was further enhanced by incorporating temperature-dependent dielectric properties by Liu et al. [40]. Even though with only electromagnetic and heat transfer physics considered, the models would be capable of predicting the temperature distribution within the oven cavity and the food loads. The simulated results showed slightly higher temperatures than the experimental ones, which might be due to the neglection of the moisture evaporation in a short-time heating process. 

Even though the models that only couple electromagnetic field and heat transfer physics cannot predict the heating results with very high accuracy, they can still be used to guide the further improvement of the heating process. Two of the most widely accepted strategies applied for better heating uniformity, turntable, and mode stirrers, are vastly simulated with 3-D models for a better understanding of the interactions between the nonstationary electric field and the target food products [17,22,24,41,55,57]. The models were also developed and used to explore the potential advantages of different improvement approaches, such as using multiple microwave ports [42], optimization of the waveguide geometry design [43], and mode stirrer made of multiple materials [23].

In addition to modeling microwave heating in commercial domestic microwave ovens, simulation of electromagnetic heating was also performed for the solid-state microwave systems that are not commercially available but still under development. The models aimed to reveal the relationship between microwave parameters (frequency, power, and relative phase) and the heating performance to better design the solid-state microwave systems. The solid-state microwave system has the advantage of precise and flexible control over microwave parameters, including frequency, power, and relative phases (for multiple ports system). The electromagnetic heating modeling work by Tang et al. [44] demonstrated a frequency-selection method to improve the microwave heating uniformity. By simulating the heating process under different frequencies, the frequencies that generated high power efficiency and low reflection were determined to improve the heating uniformity and the power efficiency. Asides from frequency selection strategies, the frequency-shifting rate was also investigated. Through the 3-D electromagnetic heating models, Du et al. [13] further explored the key factors that affected the shifting-frequency approach. The simulation results showed that both the frequency shifting step and rate would influence the heating performance significantly, and therefore, careful design of the shifting strategy is critical for improving microwave heating performance. Moreover, a novel cavity structure with a movable oven wall that can change the phase of the input microwaves by shifting the wall was also simulated with this type of mechanical model by Liao et al. [45]. By simulation and experiments, a phase-shifting strategy was developed to improve the heating uniformity by up to 58%. 

#### 2.3.2. Electromagnetic Heating and Mass Transfer

Although the electromagnetic heating models that coupled electromagnetics and heat transfer had shown their usefulness in enhancing the understanding of interactions between microwave and food products, the model predictions accuracy need to be improved, especially for a longer time heating process, where the evaporation and transport of moisture play important roles in energy balance and heat transport inside the food products. Therefore, several comprehensive modes that incorporate electromagnetics, heat transfer, moisture evaporation, and mass transport were developed. 

Gulati et al. [46] and Zhu et al. [47] developed multiphysics-based mechanistic models that simulated the microwave drying of spherical potato samples where the moisture loss in food cannot be ignored. Different from the electromagnetic heating models where foods are considered as single-phase material (solid or liquid), the potato samples were taken as porous media products that consisted of solid (skeleton), liquid (water), and gas (water vapor and air) phases, which can be mathematically described as following equations:

(14) $$\Delta V=\Delta V_{s}+\Delta V_{f}$$

(15) $$\phi =\frac{\Delta V_{f}}{\Delta V}=\frac{\Delta V_{w}+\Delta V_{g}}{\Delta V}$$

where *s* and *f* are the solid and fluid phases, respectively, *V* is the total volume of the material, and ϕ is the porosity. 

In the comprehensive models, Darcy’s law was used to solve the momentum conservation of different phases, which describes the flows of different phases due to the gradients in gas pressure within the material:

(16) $$\hat{v_{i}}=-\frac{k_{in,i}k_{r,i}}{\mu_{i}}\nabla P$$

where *i* = *w*, *g*, denoting the liquid water and gas phase, respectively, $k_{in,i}$ and $k_{r,i}$ are the intrinsic and relative permeability, respectively, μ is the dynamic viscosity, *P* is the gas pressure. 

The mass conservation of phases was calculated as:

(17) $$\frac{\partial c_{w}}{\partial t}+\nabla \hat{n_{w}}=-\dot{I}$$

(18) $$\frac{\partial c_{g}}{\partial t}+\nabla \hat{n_{g}}=\dot{I}$$

(19) $$\frac{\partial c_{v}}{\partial t}+\nabla \hat{n_{v}}=\dot{I}$$

where $\dot{I}$ denotes the volumetric source term due to phase change and *c* is the concentration of each phase, and $\hat{n}$ is the unit normal of each phase. The mass flux for each phase can be expressed as follow:

(20) $$\hat{n_{w}}=\rho_{w}\hat{v_{w}}-D_{w,cap}\nabla c_{w}$$

(21) $$\hat{n_{g}}=\rho_{g}\hat{v_{g}}$$

(22) $$\hat{n_{v}}=\rho_{v}\hat{v_{g}}-\left(\frac{C_{g}^{2}}{\rho_{g}}\right)M_{v}M_{a}D_{bin}\nabla \chi_{v}$$

where $D_{w,\mathrm{cap}}$ is the capillary diffusivity, $D_{bin}$ is the vapor diffusivity, $M_{v}$ and $M_{a}$ are molecular weight for vapor and air, $C_{g}^{}$ is the molar density, $\chi_{v}$ denotes the mole fraction of vapor in gas phase.

With the phase change and movement of phases along with microwave power absorption, the thermal energy balance of the heating process was calculated as:

(23) $$\rho_{eff}C_{P,eff}\frac{\partial T}{\partial t}+\underset{i=w,v,a}{\sum }\left(\hat{n_{i}}\nabla \left(C_{p,i}T\right)\right)=\nabla \left(K_{eff}\nabla T\right)-L\dot{I}+q$$

where $\rho_{eff}$, $C_{P,eff}$, and $K_{eff}$ are obtained by averaging the values of solid, liquid, and gas phases, weighted by either mass or volume fractions. In the equation, *a* denotes the air domain, and L is the latent heat. 

The comprehensively developed model was able to predict the time-dependent spatial power, temperature, evaporation, pressure, and moisture distribution within the sample, which allowed the users to understand the microwave heating process more comprehensively than the electromagnetic heating models. For example, by comparing microwave drying of samples at different sizes, the simulation results demonstrated the importance of mass transfer in a small sample when heated with microwave power. 

However, the previously mentioned model did not include the rotational turntable that is already widely used in domestic microwave ovens. Chen et al. [48] built the heat and mass transfer model with the rotational process included. Homogenous mashed potato samples were used for simulation and validation. Since many parameters (e.g., evaporation constant, intrinsic permeability diffusivity, etc.) cannot be obtained from literature or measurement, a sensitivity analysis was performed on the input parameters, and the modeling results implied that intrinsic gas permeability and the water diffusion coefficient had little influence on the simulation results. The conclusion could help properly determine the most significant parameters for a reliable model. Another porous material heating model by Pitchai et al. [49] that simulated the rotational microwave heating of a heterogeneous sample (lasagna) was developed and validated. The heterogeneous lasagna sample with 6 layers, composed of meat sauce, pasta, ricotta cheese, pasta, meat sauce, and pasta from top to bottom layer, was used as a model food. Furthermore, the heating scenario was started from frozen temperature (−10 °C), making the model more realistic since most ready-to-eat microwavable meals are available as frozen products. The good agreement, in terms of transient temperature and total moisture loss, between the simulation and experiment results made it a useful tool for further exploration of the potential strategies to improve the heating performance that cannot be simply done with experiments alone, such as the package shape, layout, food geometry or ingredients for each layer.

#### 2.3.3. Electromagnetic Heating and Structure Deformation

When foods with high porosity whose structure change significantly due to both the high internal pressure and the moisture loss are heated in a microwave oven, the material deformation of the sample should be coupled in the model as it alters the interaction with microwaves, and as a consequence, modifies the heating results [58]. The internal heating at a fast rate by microwave generates evaporation and pressure, causing volumetric shrinkage induced by moisture loss and puffing induced by increased internal pressure. The final structure change is determined by the main contributor from these two elements. In a general meal heating process, considering the relatively large food volume, the moisture loss contributed primarily to the volume change, and hence usually, the drying results in a reduced volume [50]. A 3-D mechanistic model by Gulati et al. [50] coupled electromagnetic physics, heat and mass transfer physics, and solid deformation physics to simulate the microwave drying of potato samples. The solid domain of the material was treated as hyperelastic and included in the model using the Arbitrary-Lagrangian-Eulerian (ALE) framework, a powerful tool to handle the Fluid-Structure-Interaction problems in FEM models [59,60]. The ALE framework considered the mesh movement of the fluid domain, such as air, caused by structure change of the solid domain, the skeleton of the dry material. In order to control the degrees of freedom in the model, the ALE framework was only applied within the food domain to make the problem solvable. In the drying of a small sample (e.g., potato cube in 10 cm × 10 cm × 10 cm), over 20% volume loss was observed, which can barely be ignored. In order to describe the deformation of the material structure, two driving forces were considered: (1). moisture loss; (2). gradients in gas pressure arising due to internal evaporation. The volume change due to moisture loss, $J_{M}$, is obtained by the change of the liquid water within the material as:

(24) $$\Delta V\left(1-\phi_{w}\right)=\Delta V_{0}\left(1-\phi_{w,0}\right)$$

(25) $$\frac{\Delta V}{\Delta V_{0}}=J_{M}=\frac{1-\phi_{w,0}}{1-\phi_{w}}$$

where 0 in subscript denotes the initial value for the parameter. The deformation gradient due to moisture loss, $F_{M}$, is:

(26) $$F_{M}=I_{M}I$$

I is the identity tensor. 

To calculate the gas pressure-induced deformation:

(27) $$\nabla_{Χ}\cdot \left(S\prime \prime F_{el}^{T}\right)=\nabla P$$

(28) $$S\prime \prime =I_{el}F_{el}^{-1}\sigma \prime \prime F_{el}^{-T}$$

where S″ is the second Piola-Kirchhoff stress tensor, σ″ is the Cauchy stress tensor. 

The corresponding volume change $I_{el}$ is:

(29) $$I_{el}=det\left(F_{el}\right)$$

And with the above, the overall deformation gradient F follows:

(30) $$F=F_{el}F_{M}$$

Then, the combined, time-dependent volume loss ratio can be expressed as

(31) $$I=det\left(F\right)$$

And the time-dependent porosity $\phi \left(t\right)$ follows:

(32) $$\phi \left(t\right)=1-\left(1-\phi_{0}\right)\frac{\Delta V_{0}}{\Delta V}=1-\frac{1-\phi_{0}}{I}$$

The validation experiment results showed that with the shrinkage included, compared to the model without deformation considered, the prediction accuracy for both temperature profile and moisture change was improved. By sensitivity analysis by the validated model, the elastic modulus value of the material was critical to the moisture loss and volumetric shrinkage of the samples, implying a necessity to precisely measure the elastic modulus value as model input if the material shrinkage contributes a lot in the process. 

#### 2.3.4. Electromagnetic Heating with Chemical and Microbiological Kinetics

Aside from the physical dimension change during microwave heating, the chemical compositions of the food product, especially the heat-sensitive ones (e.g., antioxidants, vitamins), also degrade with heating time and temperature, and thus the chemical kinetics can be incorporated into the mechanistic models to understand the chemical degradation process. Theoretically, any kinetics that can be quantitively described can be coupled to the mechanistic models, and the accumulative effects of temperature and heating time can be obtained. The kinetics in food processing usually follows zero-order (e.g., decomposition, Equation (33)), first-order (e.g., degradation, Equation (34)), or second-order (e.g., change of amino acids involved in Maillard reaction, Equation (35)) reaction models [61].

(33) $$-\frac{dc}{dt}=K$$

(34) $$-\frac{dc}{dt}=Kc$$

(35) $$-\frac{dc}{dt}=Kc^{2}$$

where c is the concentration of the objective compound, K is a rate constant. 

To induce temperature-dependent variables, Arrhenius’ law also can be integrated as Equation (36):

(36) $$kK=Ae^{-\frac{E_{a}}{RT}}$$

where A is the pre-exponential factor, E_a_ is the activation energy, and *R* is the gas constant. 

The coupled kinetics models are then coupled with the established multiphysics-based models to simulate thermal-activated dynamic kinetics, such as color change [51,52], vitamin degradation [53] and polyphenol decomposition [52]. 

Microbiological kinetics also have been coupled to the mechanistic models to predict thermal inactivation efficacy. To assess the volumetric microbial inactivation effect, a 3-D model developed by Roohi and Hashemi [54] evaluated the average volumetric temperature of carrot slices by microwave heating simulation at different power levels (200, 400, and 600 W) and determined the corresponding inactivation effects on different types of microorganisms. The assessments, based on temperature profiles and processing time, were conducted in three phases: heating process, holding temperature, and microwave power-off phase. The simulation results showed that, at 600 W, most inactivation occurred in the first two phases; and at 200 W, the temperature was not high enough to product inactivation after the last phase. And for 400 W heating, the highest inactivation occurred during the last phase. Besides, compared to the conventional pasteurization method using steam, the energy consumption by microwave was reduced by 52.3%.

Because of the thermal runaway phenomena in frozen foods, the temperature of the cold spots of the foods can be too low to kill the microbes, and these undercooked areas tend to leave the microbes to flourish [62,63]. Hence, it is also necessary to evaluate the pasteurization/sterilization effects over different regions within the food. The model by Pitchai et al. [55] evaluated the processing time needed to achieve desired 7 log reduction of *Salmonella* at centers of chicken nuggets placed at different locations in the oven cavity. The results implied that a typical 90 s heating of the frozen chicken nuggets from −5 °C was not enough to completely achieve the sterilization results at all locations; and among all chicken nuggets, the one placed in the center only reached 35 °C and could never reduce the microorganisms by 7-log. The simulated spatial change of microbiological compounds inside the sample through 3-D mechanistic models can better describe the dynamic alteration and regional difference of the objective compounds and can be applied to optimize the layout of the food system for better inactivation.

### 2.4. Model Implementation Considerations

**Figure 1:**
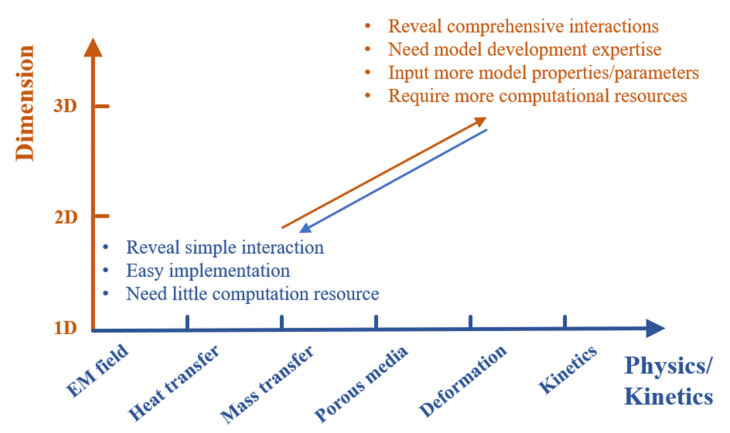
Development of mechanistic models with combinations of dimension and physics/kinetics.

Within these mechanistic models for modeling the physical, chemical, and biological processes during microwave heating, geometric dimensions and physics/kinetics are the two major factors that influence their development and applications, as shown in Figure 1. The simple model with 1-D geometry and basic physics of electromagnetics can help the users briefly understand the microwave heating process; and these models only need little computational resources (computation time and computation system) for simulation and are easy to be implemented. In contrast, the comprehensive models that incorporate both detailed 3-D geometries of oven and food and comprehensive physics and kinetics are more powerful for the users to fully understand the complicated interactions among microwaves, foods, and packages. Meanwhile, these models also need more model inputs (model parameters, material properties, etc.) and more computational resources to generate meaningful results. When oven and food developers develop and implement the mechanistic models in their product design, it is better to balance the inputs (computational resources, expertise in model development, model parameters and properties) and outputs (model accuracy, efficiency, and usefulness) and select the proper combination of dimensions and physics/kinetics. The strategy for physics/kinetics coupling also influences the model accuracy and efficiency, which needs to be considered during the model development.

#### 2.4.1. Dimensions

The simple 1-D models were instructive in understanding the microwave heating process. The relatively simple implementation allows fast computation, which is more intuitively meaningful when only a rough result is needed, such as total heat absorption or overall power absorption efficiency that can be obtained based on the whole domain. While compared to the comprehensive models that incorporate detailed 3-D geometries, the low dimensional models have apparent drawbacks. The 1-D model can hardly be validated through experiments, which causes doubt on the robustness of such models; and quite many assumptions are made in the calculations, such as homogeneous parameters without region-wise analysis. Therefore, the 1-D models can barely be used to understand the spatial uniformity problems.

Furthermore, the 2-D models are more meaningful than the 1-D models since the 2-D geometries can convey spatial information over a layer and have been used to analyze the temperature or power distribution. However, similar to 1-D models, the simulation results from 2-D models cannot reflect the microwave heating phenomenon in real life since the electric field and microwave power are distributed non-uniformly both vertically and horizontally. With the benefits and limitations, the 2-D models are suitable for simulating some simplified problems where the thickness of the sample is thin enough [7] and scenarios that symmetric conditions can be applied (e.g., cylindrical sample) [16,38,64,65].

Along with the development and application of more powerful computational resources, 3-D models are able to simulate and visualize 3-D spatial results, which reflects the real microwave heating process. Compared to the 1-D and 2-D models, the detailed 3-D geometries of the oven cavity and food product are usually incorporated in the model and have been proved critical for accurate prediction of the results. However, these geometry details require fine meshing elements and cause significantly expensive computational resources. The capability of predicting 3-D spatial results also challenges the validity of the model results. In the 3-D model validation, the accurate prediction of spatial values is expected to be achieved. For example, the transient point temperatures were often measured by fiber optic sensors to validate the temperature change during the whole heating process. However, accurate prediction is difficult because it is not only important to incorporate accurate geometry details in the models but also critical to use proper governing equations (physics and kinetics) to accurately describe the microwave heating processes.

#### 2.4.2. Physics/Kinetics

The other key factor that needs to be considered in model development is the physics/kinetics to be incorporated. On the one hand, more physics/kinetics could enable food developers to understand the microwave heating process more thoroughly. On the other hand, more physics/kinetics also need more model inputs of material properties, parameters, and computational resources (time and system). The selection of physics/kinetics should be decided based on the specific applications and balance between inputs and outputs. For example, in a short microwave heating process where the physical process does not change much (e.g., no apparent evaporation), the simple electromagnetic heating models might be sufficient to understand the microwave-food interactions [17,55,66]. These models could provide useful information on how the microwave power is spatially distributed at hot and cold spots and how heat transfers within the food product. However, in a long heating process where moisture evaporation is significant, the neglection of moisture evaporation and mass transfer could lead to increased simulation errors [40]. Moreover, comprehensive physics may be necessary for other applications where a significant non-linear process occurs. For example, in microwave-assisted drying, puffing, and frying, the moisture content and structure of the product domain change significantly with time, the electromagnetic heating with mass transfer and structure deformation models are essential to understand these processes. 

#### 2.4.3. Coupling Strategy

**Figure 2:**
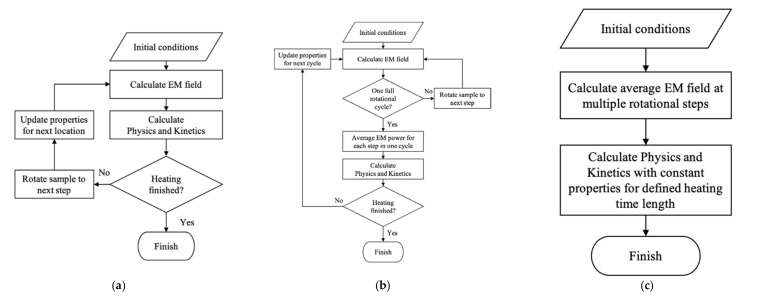
Physics coupling strategies for multiphysics mechanistic models. (**a**) rotational step coupling; (**b**) rotational cycle coupling; (**c**) and decoupling strategies.

Microwave heating is a non-linear process that involves the rotation of food products, properties change with temperature, and the coupling of multiple physics/kinetics (electromagnetic field and other physics/kinetics). The incorporation of these non-linearities into the model influences the model efficiency and accuracy significantly. In a rotational microwave heating model, the rotation of food products was often considered as discrete rotational steps due to the limitation of simulation capability in dealing with Maxwell’s Equations. The material properties are changing continuously with time and temperature that need to be updated simultaneously with the rotations. The electromagnetic field simulation is also separated from other physics/kinetics simulations. There are three types of coupling strategies used for modeling the microwave heating of foods with location rotation, properties update, and physics/kinetic coupling, as shown in Figure 2, namely rotational step coupling, rotational cycle coupling, and decoupling.

The rotational step coupling strategy (Figure 2a) was the commonly used approach that simulated the electromagnetic field, updated the temperature-dependent dielectric properties, and simulated other physics/kinetics at each rotational step during the whole microwave heating process [53]. The frequent properties-update requires to switch the solvers between electromagnetic field simulation the other physics/kinetics simulation back and force, which increases the computation time.

Several studies proposed strategies to evaluate and improve the computational efficiency of the microwave heating models. Considering the fact that the dielectric properties may not change much during a short heating time (e.g., one rotational cycle), Chen et al. [67] evaluated the approach of updating dielectric properties for one rotational cycle (Figure 2b) instead of traditionally used updating for one rotational step. In this rotational cycle coupling, the electromagnetic fields at multiple locations during one rotational cycle were simulated using the same dielectric properties first; then, the averaged electromagnetic field of the multiple locations was calculated and used for the simulation of other physics/kinetics (e.g., heat transfer, mass transfer, etc.) for one rotational cycle; after that, the dielectric properties were updated for next rotational cycle simulation. The simulation results found that the rotation cycle coupling strategy could reduce the computational time by 83% without considerably influence the model accuracy.

Chen et al. [68] further demonstrated a decoupled multiphysics modeling strategy to reduce the computation time, as shown in Figure 2c. Instead of updating the dielectric properties either at each step or each cycle during the microwave heating process, the decoupled strategy demonstrated that the electric field could be simulated using room-temperature dielectric properties throughout the whole modeling process without influencing the model accuracy much. The comparison between the coupled (rotational cycle coupling) and decoupled models implied that the decoupling methods would reduce the computation time by about 90% while sacrificing very little of the simulation accuracy. However, the decoupling strategy is only suitable for the high moisture and low salt content food products, where the dielectric properties do not change significantly [69].

In general, each type of model with various geometric dimensions and physics/kinetics has its advantages/disadvantages for specific research purposes. The key is to properly select dimensions, physics/kinetics, and coupling strategies to solve specific problems.

### 2.5. Model Validation Strategy

In order to assess the performance of the model prototypes before using them for further research/product development, validation experiments need to be performed to confirm the model accuracy. Usually, a well-developed model needs to be validated through more than one validation method since each method suffers from unique systematic errors.

The top surface temperature comparison is one of the most commonly used approaches, where electromagnetic heating, moisture evaporation, and conductive and convective heat transfer can all influence the heat pattern. In short-time microwave heating scenarios, where the temperature of the hot spot is much lower than the boiling point of water, the basic model with electromagnetics and heat transfer physics can have a good validation result if properly established [17]. While in longer heating scenarios, where the moisture movement takes place, the simulated top surface temperature tends to be higher than the experimentally measured ones [13,40,53,67,68]. The potential systematic error of this approach of comparing top surface temperature that results in the temperature difference between simulation and experiment comes from the operational delay, which denotes the time-lapse between the end of the heating and thermal image capture [70].

Internal temperature profiles of the sample can also be captured by a thermal camera after heating for validation. Due to the unique heating principle of microwaves, the inner heat distribution differs from the surface and can be used for model validation [9,38,70,71]. The general problem with the inside layer thermal image validation is that due to the time-lapse, the non-uniformity of the pattern distribution declines. But whether the locations of the hot and cold spots are properly predicted is one of the critical evaluation criteria for the model performance.

In order to complement the validation with only the final temperature, transient point temperatures recorded with several optical fibers through the whole heating process are used to compare the temperature at critical locations, such as the corners and center of the sample, which are more likely to suffer from overheating and underheating in microwave heating. This validation strategy collects more information and suffers less from the time delay issue. However, the potential problem of this method is the unexpected movement of the fibers during heating [40,67,70]. Due to the mass transfer within the sample and the shape change at the corners or near the edges of the sample, the fibers can hardly be fixed during heating, which can lead to failure in transient temperature validation. While since the movement is usually slight, the temperatures measured at different locations can still be used to validate the relative locations of hot and cold spots for illustrative purposes.

In addition to thermal profiles, in models that simulate the heating process of foods with high moisture or in small volume size, where the mass transfer can make a great difference to the final results, the moisture change due to the microwave power should also be validated. The total moisture loss [3,46,49,50,67,72,73] and/or transient point moisture content [74] have been measured for validation. Besides, the pressure generated due to moisture evaporation was also measured for validation [46]. In microbial kinetic coupled models, the validation can also be conducted by cell culture to assess the prediction of the sterilization effects [55,75,76,77].

### 2.6. Other Mechanistic Models Applied in the Non-Domestic Oven and/or Non-Food Research

Aside from the application in domestic ovens, the microwave is used in customized lab-scale or industrial-scale microwave systems for pasteurizing foodborne pathogens. Mechanistic models have been developed to simulate and understand the microbial inactivation processes. Several 3-D mechanistic models coupled with inactivation kinetics were developed by Hamoud-Agha et al. [75,76] to assess the microwave inactivation on *E. coli* in calcium alginate gel using a customized lab-scale microwave system. The models investigated the influence of different holding times during the heating process and found that the holding pause (regular on-off the power) did not help improve the temperature uniformity within the sample and hence could not assist in improving the microbial inactivation performance. Another mechanistic model based on a customized lab-scale microwave system by Albuquerque et al. [77] simulated the microwave pasteurization of in-package ground beef products and showed that with a lower microwave heating rate, the inactivation performance was better. There was a negative correlation between heating rate and pasteurization result, which could be attributed to better temperature uniformity generated by the slower heating process.

As a high-efficiency thermal treatment technique, microwave heating is also widely used in many other fields, such as material processing and chemical treatment. Similar mechanistic models were developed to simulate the microwave heating of these non-food products. Several mechanistic 1-D models by Monzó-Cabrera et al. [78,79] that coupled electromagnetic, heat and mass transfer physics simulated the microwave drying of leather material where the moisture loss was critical in the process and could not be ignored. Similar models of the microwave drying process, while on laminar materials, were proposed by Monzó-Cabrera et al. [80,81,82] to estimate the moisture content and heating efficiency of the drying process. A 2-D model by Funawatashi and Suzuki [83] simulated the microwave heating of ceramic material in an oven cavity, with electromagnetic and heat transfer physics included, and revealed the heat distribution over a horizontal plane. Another 2-D model by Ciacci et al. [84] was implemented with electromagnetic, and heat and mass transfer physics to simulate the microwave drying of woodblocks. Holmes et al. [85] implemented a 3-D mechanistic model to study the thermal change by microwave during a chemical reaction. 

Sharing similar principles, the microwave heating models applied in either food-related research or non-food fields are employed to reflect the complicated microwave-material interactions. As an important means of heating, on both lab and industrial scales, microwave heating keeps gaining popularity. The mechanistic models based on physical, chemical, and biological processes are playing critical roles in assisting the development and application of microwave techniques.

## 3. Machine Learning Models

In addition to the mechanistic models that were developed based on well-established physics and kinetics, machine learning is an emerging technique in modeling the microwave heating process. There are two approaches that machine learning can be used: one is to couple machine learning with the mechanistic model as a hybrid model; the other approach is to develop a data-based pure machine learning model. 

### 3.1. Hybrid Model That Couples Machine Learning with Mechanistic Models

The machine learning technique has been used as a powerful tool for big data analysis [86]. Since the multiphysics-based mechanistic models have the ability to extensively simulate various conditions, a well-implemented and validated model could generate a large amount of data, making the data analysis also vital to the modeling process. There is an increasing trend to use the hybrid modeling strategy that integrated machine learning models with the multiphysics models to better analyze and understand the results generated from simulation scenarios, as well as to optimize the microwave heating process. With the multiphysics-based simulation model serving as a data generator and the machine learning model functioning as an analyzer, the simulation model provides more informative data to the learning model than the manual experiment does, and in turn, the learning model enhances the utilization of the large dataset than the manual analysis does.

**Figure 3:**
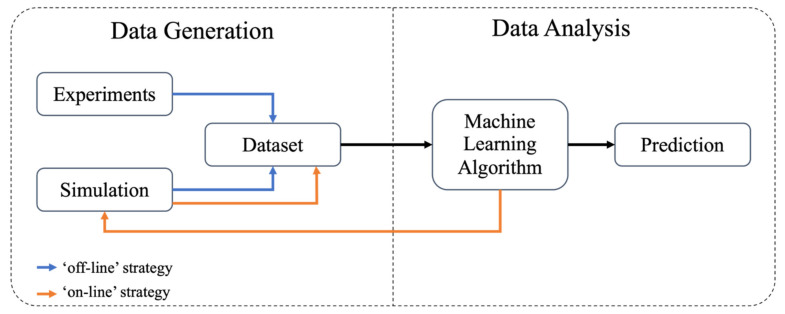
The on-line and off-line strategies that integrate machine learning with mechanistic models.

There are two types of hybrid models depending on how the machine learning and mechanistic models are coupled, namely off-line and on-line, as shown in Figure 3. The key difference between these two types of models is the training dataset. In the off-line model, the training dataset only inputs one group of mechanistic model results without updating, while the on-line model updates the training dataset by inputting more data during the learning process and formed a loop with the mechanistic model [87]. The on-line hybrid model loop starts with providing limited simulated results (x~f(x)) from the mechanistic model to the machine learning model. The machine learning model uses these initial data as a training dataset, analyzes the data, and also guides the mechanistic model for further simulation. After that, the newly simulated results can be used to expand the training dataset for further training, analysis, and guidance. The loop between mechanistic modeling and machine learning will end when the optimization process converges after meeting the predefined stopping criteria (e.g., upper limits for the number of the optimization steps, lower limits for result improvement).

#### 3.1.1. Off-Line Coupled Hybrid Model

An off-line hybrid model, namely gradient descent, was developed and applied to optimize the rotation step of a cutoff part over the waveguide of the microwave oven by He et al. [88]. The spatial temperature profiles at different discrete rotational angles were simulated by the multiphysics model and fitted to a linear regression model. The gradient descent algorithm was used to optimize the time step for each angle to achieve the best heating uniformity. The optimized rotation strategy developed based on integrated multiphysics modeling and machine learning approach was validated by experiments on samples in different materials, shapes, sizes, and locations inside the oven. A better heating uniformity was obtained with the optimized heating algorithm, and the experiment results confirmed the feasibility of this gradient dissent optimization strategy.

A more complicated hybrid model that integrated multiphysics-based mechanistic models and single-layer artificial neural network (ANN) by Yan et al. [89] explored the influence on the power reflection (S-parameter) by modifying the geometry design of the microwave passive components. In this hybrid model, all the collected data from model simulation were divided into training, validation, and test datasets. The trained ANN model showed high accuracy in electromagnetic behavior prediction with respect to the geometry parameter inputs. The CPU time for predicting a test sample by using a trained machine learning model, compared to the multiphysics model, was reduced by 98%. Given the characteristic of automatic hyperparameter selection and updating, the hybrid model could be used without users’ understanding of the model structure or predefined parameters [90].

Another off-line hybrid model was developed based on the coupling of multiphysics models and convolution neural network (CNN). Lähivaara et al. [91] implemented the hybrid model to predict the moisture distribution in samples treated with microwave heating. By simulating the microwave heating process through multiphysics modeling, the S-parameters were recorded as inputs for the CNN model. The trained model showed good performance in prediction, with a fast calculation speed due to its pattern recognition ability. And in this research, the CNN model also demonstrated its advantage in handling high-dimensional parameters, where both input and output values can be multi-element vectors, making it feasible to directly predict spatial results.

#### 3.1.2. On-Line Coupled Hybrid Model

The on-line hybrid model is the other approach that uses a two-way coupling strategy to integrate the mechanistic model and machine learning model in a loop to achieve optimization of the microwave heating process. Yang et al. [69] developed and used an on-line hybrid model to optimize the thickness of frozen microwavable food by assuming the non-linear relationship between the food geometry and the heating results. The hybrid model that coupled mechanistic simulation and non-parametric Gaussian Process regression with the hyperparameters optimized by the Bayesian optimization algorithm was developed. Comparing to the traditional trial-and-error simulation strategies, the proposed hybrid model could reduce the number of simulation models by around 60% with the on-line learning strategy. The optimized thickness could have even better heating results compared to the one obtained from trial-and-error methods.

### 3.2. Pure Machine Learning Models

Besides the hybrid models, there is also a tendency to use machine learning models alone to analyze the relationship between experimental parameters and the microwave heating results, which does not require any mechanistic models but only experimental data. As long as a specific objective is known, it is possible to develop such numerical models for prediction or classification purposes. The most used model is the deep learning model, where the relationship is taken as a black box, and no specific parametric models need to be nailed down. Dash and Das [92] implemented an ANN-based model to predict the microwave puffing performance. Four parameters (microwave power, processing time, and concentrations of two key ingredients) were used as inputs, and the expansion ratio and the percentage of puffed samples were used as outputs. The finalized bias and weight parameters from the well-trained ANN model were extracted and used as input to a genetic algorithm (GA) for predicting puffing parameters and optimizing the combination of the four input parameters. The integrated ANN-GA model had very high prediction accuracy and was able to provide very detailed precondition parameters for the sample preparation.

Another deep learning model by Wang et al. [93] applied a high-dimensional input dataset collected by measurement to training a CNN model for feature extraction and then an unsupervised learning method, named isolation forest algorithm (IFA), to detect the presence of local overheating from the features. In this study, the input dataset for each sample point included some initial conditional parameters, such as initial temperature and input power level, and some dynamic parameters obtained by real-time measurements, such as transient temperature and reflected power. The output that indicated the location of the overheated spots from the deep learning model with CNN was more accurate than that from the model without CNN. The prediction accuracy expressed as AUC in this study could be improved from 0.53~0.76 to 0.78~0.84 by integrating CNN. Thus, the deep learning model could be applied to better discover the overheating and assist in developing an immediate problem-solving strategy.

Both the hybrid model and the pure machine learning model can largely save the model simulation time compared to the approach with only mechanistic models. However, since the machine learning models are trained with only selected parameters, they are not compatible with extra parameters, which makes the established model less flexible to other add-on parameters, if any. The models must be re-trained if new parameters are considered, which makes this strategy less applicable than the mechanistic simulations.

### 3.3. Other Machine Learning Models Applied in Microwave Heating of Non-Foods Research

The integrated mechanistic and machine learning modeling strategy is also applied to microwave treatment of non-food materials. The integrated model that simulated the heating process of clay samples by Domínguez-Tortajada et al. [94] used GA to optimize the multi-layered dielectric material structure over the clay sample for optimal heating uniformity. Another model with marble block as sample by Domínguez-Tortajada et al. [95], which was also optimized by GA, proposed the design of an oven cavity with four waveguides with optimal locations. The distribution of the electric field within the proposed oven was significantly more uniform compared to the traditional one with only one waveguide. Another integrated model by Guan et al. [96] utilized deep neural networks (DNNs) to discover the non-linear relationship between the mechanistic model inputs (geometrical parameters) and outputs (S parameters). The machine learning technique is also powerful in terms of design optimization. Mahouti [97] implemented a multi-layer perceptron (MLP) model that was coupled with an electromagnetic mechanistic model to optimize the antenna design for achieving optimal S parameters. The proposed method could largely improve the simulation efficiency.

## 4. Summary and Future Prospects

In order to address the non-uniform heating issue in the domestic microwave heating process, extensive mechanistic, machine learning, and hybrid modeling works have been done. In this paper, different types of models, including models with different dimensions and physics/kinetics, that simulated the microwave heating process were reviewed. Various strategies aiming to improve the heating performance were proposed and demonstrated. Although the models showed great usefulness in understanding the complicated interactions among microwaves, foods, and packages, few of these approaches are really applied to the development of domestic microwave ovens or the design of microwavable food products on an industrial scale. The limitation on the industrial implementation was mainly attributed to the model accuracy, efficiency, and ease of use.

Further work is needed to address the limitation and bridge the gap between scientific research and practical application, which asks for more precise, efficient, and easy-to-use models. First, there is still room to improve the simulation accuracy of the mechanistic models. Even though the predicted results can illustrate the dynamic patterns and visualize the interactions during microwave heating, the model results still cannot be used for precise prediction. The accurate model inputs (e.g., geometry, material properties), physics/kinetics setup, and coupling strategies are needed for models with better performance.

Furthermore, given the data production advantage of mechanistic models, it is now possible to explore different potentially effective but currently unachievable approaches to improving the microwave performance, such as the new design of the heating strategies or oven and food designs, in a more efficient way. Also, the spatial output data, the quantified results can be more robust for performance assessment. By now, based on simulation results, various strategies have been proposed, some of which have been experimentally validated. Additionally, with the developed models, the hybrid models that couple mechanistic simulation and machine learning analysis are gaining popularity, where the established mechanistic models serve to generate numerical data, and the machine learning model can efficiently analyze the model outputs. Depending on the size of the output dataset, the integration strategy can be either off-line or on-line, and the type of the machine learning model is to be decided based on the complexity of the dataset. The promising integration modeling strategy has shown its advantage while still grossly underused, calling for more work in the future to further reveal the power of machine learning for microwave-related research.

Another approach to promote the industrial implementation of the models is to develop them as easy-to-use applications. Since the model development needs significant expertise, the complexity in model development is a bottleneck for the food and oven developers to use in their product designs. The ready-to-use applications based on mechanistic modeling and machine learning models can unlock their potentials and enable industrial implementations.

## Figures and Tables

**Table 1:** Summary of the implementation and findings of the mechanistic models for simulating microwave heating of foods.

Dimension	Physics	Heating Scenario	Highlight	Citation
1D	Electromagnetics Heat transfer	Heat a sphere-shaped minced beef sample	Hot spots were located in the center of spheres with radii 3.5 cm.	[34]
	Electromagnetics Heat transfer	Simulate the heating scenarios of the sample with arbitrarily given physical properties and evaluated the thermal profiles along the wave propagation direction.	The simple 1-D analytical model could provide accurate simulated results compared to previously reported models.	[35]
	Electromagnetics Heat transfer	Validated through the traditional numerical model with asphalt as the objective samples.	Applying time-dependent physics properties provided more details in the simulated temperature profiles.	[36]
	Electromagnetics	Heat different food systems with two layers at equal thickness and adjusted thickness.	The two-layer food systems with adjusted thickness proposed based on the model had a better heating performance.	[37]
	Electromagnetics Heat transfer Mass transfer	Heat a mashed potato sample filled in a tray.	For simulation of the sample with a sufficiently large length and with a short heating time, constant dielectric properties can be applied without lowering the model accuracy significantly.	[32]
	Electromagnetics Heat transfer Mass transfer	Heat unsaturated materials composed of glass beads, water, and air.	In the model, frequency, particle size, and electric field intensity are important to the proper prediction of microwave drying kinetics.	[33]
2D	Electromagnetics Heat transfer	Heat slab- and cylinder- shaped potato samples	The model predicted temperature was higher than the measured results. The locations of the hot and cold spots were properly predicted.	[38]
	Electromagnetics Heat transfer	Heat a slice of deionized water sample	The model was relatively accurate in estimating the temperature field and time of hot spots over a 2D plane.	[39]
	Electromagnetics Heat transfer	Heat a 10-mm-thick sheet sample in a microwave oven with two laminar stirrers attached to the upper wall	Locations of hot spots near the sample’s edge were properly predicted, but there were some differences between measured and predicted temperature values. Lambert’s law is only applicable for materials with a high loss factor.	[7]
3D	Electromagnetics Heat transfer	Heat a brick-shaped potato sample in a microwave with the turntable on or off.	The application of a turntable can improve the heating uniformity by about 40%.	[17]
	Electromagnetics Heat transfer	Heat mashed potato samples with different sodium chloride levels, filled in cylindrical or rectangular containers in a microwave oven with the turntable on or off.	A simulation approach that simplified the step-by-step analysis of heat transfer strategy, the computation time was cut down by a one-time analysis of the initial electromagnetic fields.	[40]
	Electromagnetics Heat transfer	Heat brick-shaped potato sample in a microwave oven with a copper patch placed in the upper surface of the turntable, functioning as a mode stirrer.	The added metal patch could improve the heating uniformity and the power efficiency, and the rotation speed, size, location of the turntable influenced the effects of the proposed mode stirrer.	[24]
	Electromagnetics Heat transfer	Heat a brick-shaped potato sample in a microwave oven assembled with a brick-shaped mode stirrer rotating over the top of the sample, with the turntable on.	The incorporation of the turntable and the mode stirrer could improve the heating performance, where the turntable worked to increase the uniformity, and the stirrer worked to enhance the power efficiency.	[41]
	Electromagnetics Heat transfer	Heat a stationary potato slice in a microwave oven assembled with a rotary radiation structure, rotating at 30°/s, over the waveguide.	The rotary radiation structure could improve the heating uniformity and efficiency compared to the application of turntable.	[22]
	Electromagnetics Heat transfer	Heat water in a cylinder-shaped beaker stationarily in a microwave oven with two power ports.	The two-port microwave oven could heat the sample more uniformly and efficiently.	[42]
	Electromagnetics Heat transfer	Simulate the thermal distribution inside a rectangular-shaped traveling microwave waveguide.	The microwave energy input/output ports should be well-positioned and matched, which was significantly related to the power efficiency.	[43]
	Electromagnetics Heat transfer	Heat brick-shaped sliced potato samples were placed on a stationary bracket in a microwave oven, with a multi-component turntable rotating beneath.	The heating uniformity was increased by up to 47% by the multiple material turntable, and the material type could affect the heating results.	[23]
	Electromagnetics Heat transfer	Heat brick-shaped sliced potato samples in a microwave oven stationarily under shifting frequency.	The shifting scenarios with only pre-selected frequencies that had higher power efficiency could improve the heating uniformity and power efficiency.	[44]
	Electromagnetics Heat transfer	Heat chicken nuggets in a frequency-shifted solid-state source microwave oven.	Heating scenarios under shifting-frequency had better heating uniformity and efficiency. The heating results could be affected by the shifting rate and the shifting sequences.	[13]
	Electromagnetics Heat transfer	Heat potato slice in a microwave oven with a movable wall to adjust the microwave phase in the oven cavity.	Heating scenarios under the shifting phase with constant frequency could increase the heating uniformity and avoid the hot spot induced in the traditional stationary microwave heating method.	[45]
	Electromagnetics Heat transfer Mass transfer	Dry potato sphere in a microwave oven.	Microwave drying of spheres in larger sizes could be well simulated, while the simulation of smaller samples was less accurate in terms of gas pressure due to the low sample temperature.	[46,47]
	Electromagnetics Heat transfer Mass transfer	Heat mashed potato samples in a microwave oven.	The model was sensitive to the gas diffusion coefficient, intrinsic water permeability, and the evaporation rate constant.	[48]
	Electromagnetics Heat transfer Mass transfer	Heat multi-layered lasagna samples in a microwave oven.	Different layers of foodstuffs showed very different heating results, which could be employed as instruction for food design.	[49]
	Electromagnetics Heat transfer Deformation	Dry brick-shaped potato samples in a microwave oven.	The volume shrinkage during drying affected the power absorption, heat, and moisture transfer within the sample during heating.	[50]
	Electromagnetics Heat transfer Chemical kinetics	Thaw a cube-shaped sample in a microwave oven under either inverter or pulsed heating mode.	The novel inverter microwave heating mode made little difference in terms of color preservation and heating uniformity compared to the traditional cycled (on-off) defrosting mode.	[51]
	Electromagnetics Heat transfer Chemical kinetics	Dry semi-ripe papaya samples in a microwave oven with an intermittent heating strategy.	Reduced power ratio, defined as the ratio of microwave on time to the total cycle time (on and off), helped retain ascorbic acid content, total phenolic content, and color.	[52]
	Electromagnetics Heat transfer Chemical kinetics	Heat mashed potato samples in a microwave oven under the inverter and cycled modes.	The two modes made little difference to the heating performance in terms of power absorption, temperature uniformity, and vitamin C degradation level.	[53]
	Electromagnetics Heat transfer Microbiological kinetics	Heat the carrot slices at different power levels with the aim to inactivate different types of microorganisms.	The most inactivation occurred at the early heating stage for high power (400 W and 600 W) heating scenarios, while when heating with low power (200 W), the temperature was too low to produce an inactivation effect after the whole heating process.	[54]
	Electromagnetics Heat transfer Microbiological kinetics	Heat chicken nuggets in a microwave oven and evaluate the processing time needed to achieve desired sterilization effects.	A typical 90 s heating of frozen chicken nuggets from -5 °C was not enough to completely achieve the 7-log reduction results of *Salmonella* over the sample.	[55]

## Data Availability

The data presented in this study are available on request from the corresponding authors.

## Nomenclature

a	Air phase
A	Arbitrary amplitude of microwaves
b	Width of the oven cavity
B	Magnetic induction
c	Concentration
$C_{g}^{}$	Molar density
$C_{p}$	Thermal capacity
$C_{P,eff}$	Effective thermal capacity
d	Maximum distance measured from the sample surface
D	Electric displacement
$D_{bin}$	Vapor diffusivity
$D_{p}$	Penetration depth
$D_{w,\mathrm{cap}}$	Capillary diffusivity
e	Euler’s constant
E	Electric fields
$E_{a}$	Activation energy
$E_{el}$	Green–Lagrange strain tensor
f	Fluid phase
F	Deformation gradient
$F_{el}$	Elastic deformation gradient tensor
$F_{M}$	Deformation gradient due to moisture effects
H	Magnetic field
I	Identity tensor
J	Current flux
k	Thermal conductivity
$k_{in,i}$	Intrinsic permeability
$k_{r,i}$	Relative permeability
$K_{eff}$	Effective thermal conductivity
l	Longitudinal distance
L	Latent heat
M	Molecular weight
$\hat{n}$	Unit normal
P	Gas pressure
q	Dissipated power
$q_{0}$	Surface power
R	Gas constant
s	Solid phase
S″	Second Piola-Kirchhoff stress tensor
T	Temperature
v	Vapor phase
V	Sample volume
**Script Symbols**	
A	Pre-exponential factor
J	Total volume change
$J_{el}$	Ratio of total volum change and volum change due to moisture effects
$J_{M}$	Volume change due to moisture loss
K	Rate constant
**Greek Letters**	
α	Attenuation factor
λ	Wavelength of microwave
ε′	Dielectric constant
ε″	Dielectric loss factor
$\varepsilon_{0}$	Permittivity of free space
δ	Loss tangent
ρ	Density
$\rho_{e}$	Charge density
$\rho_{eff}$	Effective density
ω	Angular frequency
μ	Dynamic viscosity
σ″	Cauchy stress tensor
χ	Mole fraction in gas phase
ϕ	Sample porosity
